# Reply to Accelerated Silicosis—An Emerging Epidemic Associated with Engineered Stone. Comment on Leso, V. et al. Artificial Stone-Associated Silicosis: A Systematic Review. *Int. J. Environ. Res. Public Health* 2019, 16(4), 568, doi:10.3390/ijerph16040568

**DOI:** 10.3390/ijerph16071201

**Published:** 2019-04-03

**Authors:** Veruscka Leso, Luca Fontana, Rosaria Romano, Paola Gervetti, Ivo Iavicoli

**Affiliations:** 1 Section of Occupational Medicine, Department of Public Health, University of Naples Federico II, Via S. Pansini 5, 80131 Naples, Italy; veruscka.leso@unina.it (V.L.); rosaria_romano@libero.it (R.R.); gervettipaola@gmail.com (P.G.); 2 Department of Occupational and Environmental Medicine, Epidemiology and Hygiene, Italian Workers’ Compensation Authority (INAIL), Via di Fontana Candida 1, Monte Porzio Catone, 00040 Rome, Italy; lfontana73@yahoo.it

**Keywords:** silicosis, engineered stone, occupational exposure, risk assessment, risk management, safety by design, epidemic control

## Abstract

Our systematic review on artificial stone (AS)-associated silicosis and the related comment by Edwards underline the urgency to define effective strategies to assess and manage the risk of exposure to silica in workers involved in AS job tasks. Case screening programs may be important to fully understand the extent of the silicosis epidemic associated with AS and point out critical issues in workplace settings/practices that, by contributing to higher respirable crystalline exposure, favor the disease manifestation. This information may guide the identification of the most appropriate preventive measures in workplaces, especially regarding the administration of updated training and information courses, the definition of good working practices, and the application of targeted health surveillance programs. However, considering the recent epidemiological data and the severity of AS-associated silicosis, it would be appropriate (according to the hierarchy of control strategy) to also consider the application of more stringent prevention measures, such as a safety-by-design approach to the chemical formulation of the AS. Overall, the implementation of the aforementioned preventive measures should ensure an effective control of the current silicosis epidemic and, at the same time, prevent the development of new disease cases in the near future.

## Letter

Information provided by Edwards [1] and Kirby [2] concerning the occurrence of an artificial stone (AS)-associated silicosis epidemic in Australia is extremely timely and interesting. The figures they reported are of particular concern and further confirm the relevance of the data we recently published in a review on the same topic [3]. All together, these papers highlight the urgent need to define suitable strategies to properly identify, assess, and manage the risk of exposure to silica in the artificial or engineered stone industry in order to tackle the worldwide increasing cases of silicosis in workers engaged in AS job tasks.

Case-finding programs, such as that recently promoted by the Queensland Government towards over 800 AS workers, are of invaluable relevance for the detection of this disease in large exposed populations [2]. Indeed, considering the still limited (but surely alarming) evidence on this matter and the fact that most of the currently available information is derived from small, retrospective, sometimes voluntarily reported case-series, the starting point for seriously addressing this issue must certainly be based on the analysis of reliable and statistically consistent epidemiological data [3]. This is why screening programs similar to that undertaken by Queensland Government should be strongly supported and possibly promoted and encouraged worldwide. The high intrinsic value of these initiatives is not only linked to the possibility of establishing more precisely the silicosis prevalence in workers involved in the processing of AS but also derives from the opportunity to define correlations between the occurrence of disease and any shortcomings of preventive or protective measures which should be guaranteed in workplaces [2].

Indeed, as pointed out by our recent literature review [3], poorly hygienic working conditions (inappropriate workstation cleaning practices), risky working procedures (dry-cutting or drilling), lack of engineered controls (wet cutting or local exhaust ventilation) and lack of personal protective equipment (respiratory mask) may all contribute to high levels of exposure, thus favoring the occurrence of AS-associated silicosis. Therefore, by combining health screening programs with workplace survey campaigns it would be possible to obtain high quality data on AS-related silicosis that could be very useful in addressing adequate risk assessment and management strategies. For example, as administrative controls, health and safety policies in the workplace should also include updated training and information courses to be provided to AS workforce with the aim to increase awareness concerning respiratory health risks. Importantly, data extrapolated from case findings programs may be helpful in promoting awareness of occupational physicians on health risks in the AS industry and may also guide effective health surveillance programs as well as clinical management of exposed workers.

With regard to the last topic, we have read with great interest the hypothesis proposed by Edwards [1] of establishing a reasoned and evidence-based “index of exposure risk”, which may form the basis of the criteria for the application of a targeted health surveillance program using the low dose high-resolution computed tomography. From the perspective of occupational medicine, the definitions of these criteria would represent an important step forward since they would allow for a rationalization of health surveillance programs and, at the same time, would increase the possibility of an early diagnosis of silicosis. However, also considering the results observed in the sample of Australian workers that showed a decidedly high prevalence of silicosis (~24.75%; 99/400 workers), we consider that it is important to further emphasize the need for action to reduce the silica exposure of artificial or engineered stone workers.

In this regard, the hierarchy of controls, generally applied to manage chemical risks in the workplace, should also be followed in these peculiar workplace settings. According to this approach, if the substitution of a dangerous substance is not possible, then it is necessary to limit and reduce workers’ exposure as much as possible. In this context, it should be noted that AS is composed of finely crushed rocks that are mixed with a polymeric resin and its silica content is approximately 90%. Therefore, a “safety by design” approach may be applied to such peculiar chemical compositions in order to eliminate or at least significantly lower the percentage content of silica, consequently reducing the intrinsic toxicological profile of this material. This type of intervention strategy that is aimed at maintaining most of the physico-chemical properties of materials, while at the same time limiting their toxicity, has already been successfully proposed and applied to ensure the health protection of workers exposed to engineered nanomaterials [4].

Overall, stakeholders involved from academia to industry, governmental and non-governmental organizations, workers’ representatives, and occupational health and safety professionals should be effectively engaged both in a concerted response to the currently experienced silicosis epidemic and in the whole risk assessment and management process in order to minimize the development of new cases of disease in the near future.

## References

[1] Edwards G. (2019). Accelerated Silicosis—An emerging epidemic associated with engineered stone. Int. J. Environ. Res. Public Health.

[2] Kirby T. (2019). Australia reports on audit of silicosis for stonecutters. Lancet.

[3] Leso V., Fontana L., Romano R., Gervetti P., Iavicoli I. (2019). Artificial Stone Associated Silicosis: A Systematic Review. Int. J. Environ. Res. Public Health.

[4] Leso V., Fontana L., Mauriello M.A., Iavicoli I. (2017). Occupational risk assessment of engineered nanomaterials: limits, challenges and opportunities. Curr. Nanosci..

